# Eco-Gypsum Panels with Recycled Fishing NET Fibers for Sustainable Construction: Development and Characterization

**DOI:** 10.3390/ma18184305

**Published:** 2025-09-14

**Authors:** Leonardo Lima, Alicia Zaragoza-Benzal, Daniel Ferrández, Paulo Santos

**Affiliations:** 1 University of Coimbra, ISISE, ARISE, Department of Civil Engineering, 3030-788 Coimbra, Portugal; leonardolima@phd-student.uc.pt; 2 Prefecture of University Campus, Federal University of Amazonas, Manaus 69067-005, Brazil; 3 Escuela Técnica Superior de la Edificación, Universidad Politécnica de Madrid, 28040 Madrid, Spain; alicia.zaragoza@upm.es (A.Z.-B.); daniel.fvega@upm.es (D.F.)

**Keywords:** sustainable construction, precast panels, gypsum composites, recycled fishing net fibers, development, performance characterization

## Abstract

Plastic waste is currently a major environmental issue but also plays a key role in the circular economy. Recycled plastics have become suitable for use in several applications, especially in construction, where they can improve the properties of conventional materials to enable sustainable development. This study designed new eco-gypsum composites containing recycled fishing net (FN) fibers and evaluated their mechanical, hygrothermal, fire and environmental performances. All the developed composites achieved the minimum standardized strengths. Regarding the impact hardness test, the composite with 40% recycled FN fibers (FN40%) reached a five times higher energy of rupture than the reference gypsum sample. Indeed, FN40% presented better properties in general, e.g., 33% less water absorption by capillarity, 17% lower thermal conductivity and 40% less environmental impacts. Moreover, the use of these FN40% gypsum composites was modeled in an LSF partition wall, and it was predicted that they increased the thermal resistance by 4.4%, taking traditional gypsum plasterboards (Ref.) with the same thickness as a reference. These promising results allow us to conclude that it is possible to obtain eco-friendly gypsum composite panels by incorporating recycled FN fibers, satisfying the mechanical resistance requirements (flexural and compressive) and even improving their impact hardness, as well as their functional performance regarding their hygrothermal behavior.

## 1. Introduction

Plastics are among the most produced materials worldwide, with their production increasing from 1.5 Mt in 1950 to 367 Mt in 2020, and due to the global diffusion of single-use (linear-use) plastics, as well as marine litter and the release of microplastics, plastic pollution exerts high pressure on the environment [1,2]. At present, economic development takes the environment into consideration, with some policies including principles of sustainable development and the circular economy, which assumes that waste from a production process is a raw material in another one and reduces the consumption of natural resources [3]. The circular economy is a concept involving the circular use of materials by reducing, reusing and recycling them [4].

Therefore, addressing plastic waste is considered a key priority in the European Commission’s action plan for the circular economy, and together with the European Council and Parliament, they have set a new target to recycle 55% (approximately 42.4% now) of the 16 Mt of plastic waste produced annually in the European Union countries by 2030 [5]. Worldwide, only 9% of plastic waste is recycled, so it is urgent to innovate and improve recycling solutions [6]. The use of recycled plastic in construction applications can mitigate the problem of waste management, reduce costs in the building industry and make it more energy-efficient [7] by mitigating environmental impacts and reducing the need for natural resources, considering construction’s high demand for raw materials [8]. In addition, now it is essential to standardize new building techniques that minimize environmental impacts, the incorporation of polymeric waste in concrete or gypsum composites has promoted the development of new eco-friendly non-structural products for use in precast and modular construction systems [9].

In this context, recent studies have investigated the influence on the properties of gypsum-based products [10] of incorporating plastic waste, including recycled fibers from FNs [11]. FNs are considered one of the world’s biggest problems in terms of their contribution to marine pollution [12]. Plastic waste from fisheries must be reduced, and strategies aligned with the circular economy are being planned by the European Commission that encourage the recycling of FNs [13], which are mostly composed of PP, polyethylene (PE) and polyamide (PA) [14]. Furthermore, PA is the most common polymer used in fishing gear and, due to its toughness and elasticity, has found emerging applications as a recycled material in various fields, such as the clothing industry, the automotive sector and construction [15,16]. Improvements have been achieved, e.g., an improvement of 7.5% in the flexural strength [11] and a reduction of 42% in the water permeability [9], in gypsum composites with 2% and 2.5%, respectively, recycled PA6 fibers.

Although there have been studies on plastic waste, only a few of them have evaluated the influence of recycled FN fibers on gypsum’s properties. The main aim of this study is to develop new gypsum composites with progressively increasing binder replacement with recycled FN fibers and conduct a complete performance characterization by analyzing their mechanical, hygrothermal, fire and environmental behavior to verify if they achieve the current standards’ requirements for precast construction panels.

## 2. Materials and Methods

### 2.1. Materials

Gypsum powder type A, classified according to EN 13279-1:2008 [17], supplied by Sival (Leiria, Portugal), was used as a binder. As specified by the data provided by the manufacturer, its purity was greater than 90%, its bulk density was 0.6–0.7 g/cm^3^ and it had a neutral pH in a water solution.

**Figure 1:**
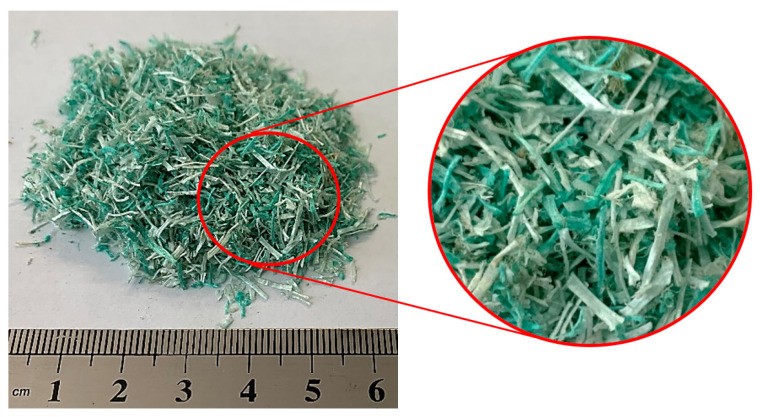
Recycled fishing net fibers.

Recycled multifilament nylon (PA) fibers were sourced from discarded waste FNs from the port of Figueira da Foz, Portugal (Figure 1). The collected FNs were washed to remove sea salt, sand and organic residues. Afterwards, they were air-dried and manually cut. The average dimensions of the recycled FN fibers were 6 mm in length; 0.8 mm in width; and 0.25 mm thick. Their bulk density was determined to be 0.21 g/cm^3^. Their thermal conductivity was 77.9 mW/m·K, determined from measurements using a λ-Meter EP500e device (Dresden, Germany), according to EN 1946-2:1999 [18].

Regular tap water, in accordance with Council Directive 98/83/EC [19], was used for gypsum hydration.

### 2.2. Sample Preparation

Samples were prepared following the technical recommendations of EN 13279-2:2014 [20]. We selected a water/gypsum (w/g) ratio of 0.7 for all the samples, as Zaragoza-Benzal et al. (2023) did in their research [21], and the proportions are shown in Table 1. This w/g ratio allowed for a plastic and workable consistency of the samples, which had a flow diameter of 165 ± 10 mm after a shaking table test. Gypsum was progressively replaced by FN fibers at a volume percentage ranging from 10% until 40%, with a 10% increment.

Firstly, the fibers and gypsum were dry-mixed before hydration to ensure homogeneous fiber dispersion and avoid agglomerations [22]. This dry mixture was sprinkled into the water for 30 s and left to rest for 1 min. The paste containing the recycled FN fibers was mixed for 30 s, then left to rest for 30 s and finally mixed again for another 30 s [20]. Afterwards, the composites were left in a controlled environment (23 ± 1 °C and a relative humidity of 55 ± 5%) for 7 days and then were dried in an oven (40 ± 2 °C) for 48 h, enough time to achieve a constant mass [20].

### 2.3. Experimental Program

The main goal of the experimental program was to characterize the newly developed composites, regarding their mechanical, hygrothermal and fire behavior. THERM (version 7.8.57) finite element software simulations were performed, and the composites’ thermal performances were compared to evaluate the benefits of the newly developed material for use in LSF partition walls. A life cycle assessment (LCA) was also conducted to evaluate the environmental suitability of precast gypsum panels with recycled FN fibers for sustainable construction.

#### 2.3.1. Mechanical Properties

The surface hardness was determined with a Baxlo Shore C durometer tester (Barcelona, Spain) according to EN 12859:2011 [23]. Five measurements per face were taken on two plane-parallel faces of 4 × 4 × 16 cm^3^ samples. We evaluated the flexural and compressive strength of the same samples according to EN 13279-2:2014 [20]. The specimens were placed in an AUTOTEST 200-10SW hydraulic press (Madrid, Spain), which applied a progressive load until they ruptured at speeds of 10 N/s for bending (3 points) and 20 N/s for compression, in accordance with previous studies [8].

**Figure 2:**
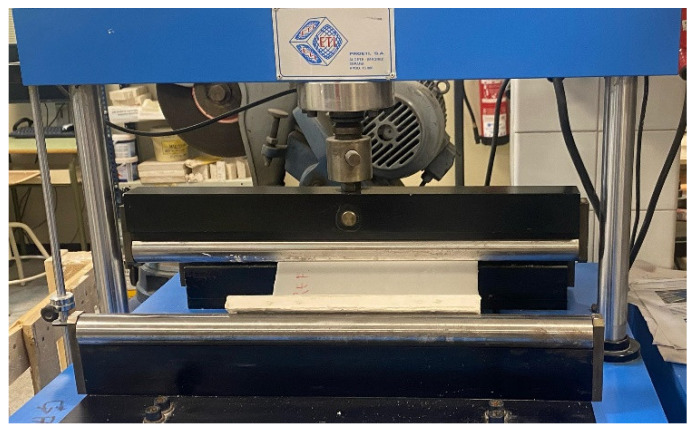
Test of plates’ flexural strength.

The flexural strength of 40 × 30 × 1.5 cm^3^ sample plates was evaluated according to EN 520:2005 [24]. The equipment used was a PÁCAM model MPX-22 machine (Madrid, Spain) with a progressive load applied until the plate’s failure, as shown in Figure 2. Afterward, an impact hardness test was performed with the same samples by adapting the EN 14158:2004 test procedure [25]. They were put on a sand bed, and a steel sphere with a mass of 1 kg was dropped from an initial height of 100 mm. If the samples did not break, the height was increased in 50 mm steps until breakage. The energy of rupture (*E*) was calculated considering the mass of the ball (*m*), the acceleration of gravity (*g*) and the breakage height (*h*) using Equation (1).

(1) E=m×g×h

A scanning electron microscopy (SEM) analysis was carried out to characterize the morphology of the samples and to establish correlations with the physico-mechanical test results. SEM observations were performed using a TESCAN Vega 4 microscope (Brno, Czech Republic) operating at 20 kV. To ensure proper conduction of the electron beam, the samples were sputter-coated with a thin gold film using a Cressington 108 auto-evaporator (Watford, UK). The analyzed 4 × 4 × 16 cm^3^ samples were extracted from the inner matrix of the gypsum composite using a hammer and a laboratory chisel in a meticulous process carried out by professionals at the Research Support Center of the Complutense University of Madrid (CAI-UCM).

#### 2.3.2. Hygrothermal Properties

**Figure 3:**
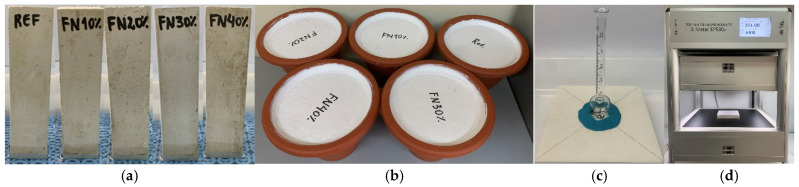
Some hygrothermal tests: (**a**) water absorption by capillarity; (**b**) water vapor permeability; (**c**) water penetration; (**d**) thermal conductivity.

The coefficient of water absorption by capillarity was determined by adopting the procedures defined in EN 1925:1999 [26]. Prismatic samples (4 × 4 × 16 cm^3^) were weighed after drying and placed vertically on a grid in water at a depth of 10 mm (Figure 3a). At time intervals of 1, 3, 5, 10, 15, 20 and 40 min, the specimens were removed from the water, lightly dried and weighed, and the heights to which they had absorbed water were measured with a caliper; then they were replaced in the container. Thermography photos were taken at the end of the test using an FLIR thermographic camera (Wilsonville, OR, USA).

The water absorption (*W*_ABS_) and open porosity (*ρ_o_*) were determined using adapted EN 14617-1:2013 [27] and EN 1936:2006 [28] test procedures. First, the dry weight (*M_D_*) of the samples (4 × 4 × 16 cm^3^) was determined. Afterward, they were completely immersed and covered by 2 cm of water for 2 h. Then, the weights of each sample in water (*M_H_*) and out of water after being lightly dried (*M_S_*) were determined. These two properties were computed using

(2) $$W_{\mathrm{ABS}}\left[\%\right]=\frac{\left(M_{S}-M_{D}\right)}{M_{D}}\times 100$$

(3) $$\rho_{O}\left[\%\right]=\frac{M_{S}-M_{D}}{M_{S}-M_{H}}\times 100$$

According to ISO 12572:2016 [29], the cup method was used to determine the water vapor permeability (p). Firstly, plastic cups were filled with a saturated KNO_3_ solution (70 mm solution depth and 20 mm of air space between it and the sample). Then, samples with a 15 cm diameter and 2.5 cm thickness were affixed onto the cup tops with silicone (Figure 3b). The test assemblies were weighed, placed in a room (at around 23 ± 1 °C and a relative humidity of 55 ± 5%) and weighed weekly for 8 weeks. The following equations were used to calculate *P*, and Table 2 defines the corresponding variables.

(4) $$W=\frac{\Delta m}{\Delta t\times A\times \Delta p}$$

(5) P=W×d

A Karsten tube was used to evaluate the water permeability according to RILEM Test Method II.4 [30]. The test apparatus was affixed onto the 15 × 15 × 2.5 cm^3^ samples with silicone rubber, then filled with water until reaching the zero gradation mark on the column (Figure 3c). As soon as 1 mL of water had penetrated the sample, the water in the tube was quickly topped up again to the zero mark to maintain a steady water pressure of around a 10 cm head of water. Measurements of the quantity of water absorbed were taken at time intervals of 5, 10, 15, 20, 30 and 60 min.

The bulk density was determined by adapting EN 12859:2011 [23]. The mass-to-volume ratio was calculated using measurements of 4 × 4 × 16 cm^3^ samples taken with a VEVOR electronic balance (Shanghai, China) and a POWERFIX electronic caliper (Shanghai, China).

The thermal conductivity was determined using a λ-Meter EP500e (Dresden, Germany) (Figure 3d) according to EN 1946-2:1999 [18], using 15 × 15 × 2.5 cm^3^ specimens wrapped with a plastic film to prevent moisture absorption. The test was performed during the 240 min after convergence, i.e., when there was less than 1% variation in the thermal conductivity over time, and the following additional parameters were set: three average test temperatures (10 °C, 25 °C and 40 °C), with a 15 °C temperature difference between the hot and cold plates.

**Figure 4:**
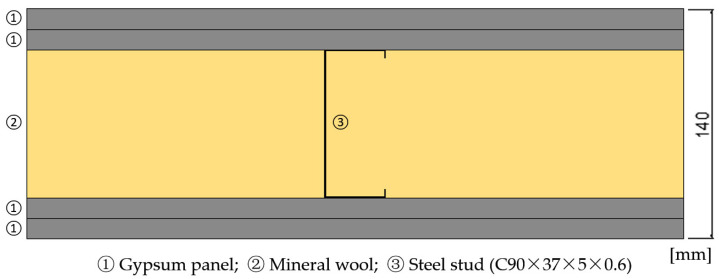
Horizontal cross-section of the evaluated LSF partition wall.

After the thermal conductivity tests, numerical simulations were performed using the THERM finite element method. A horizontal section of an LSF partition wall is presented in Figure 4, and it was modeled with a distance between the steel studs of 400 mm. The numerical computation error was capped at 2%, and a maximum of 100 iterations were set. The materials’ thickness and thermal conductivity are shown in Table 3. Regarding the boundary conditions, the interior environment temperature was set to 20 °C and the exterior at 0 °C (winter season); the surface’s thermal resistances of 0.13 m^2^⋅K/W (internal) and 0.04 m^2^⋅K/W (external) were adopted according to ISO 6946:2017 [31].

#### 2.3.3. Fire Behavior

**Figure 5:**
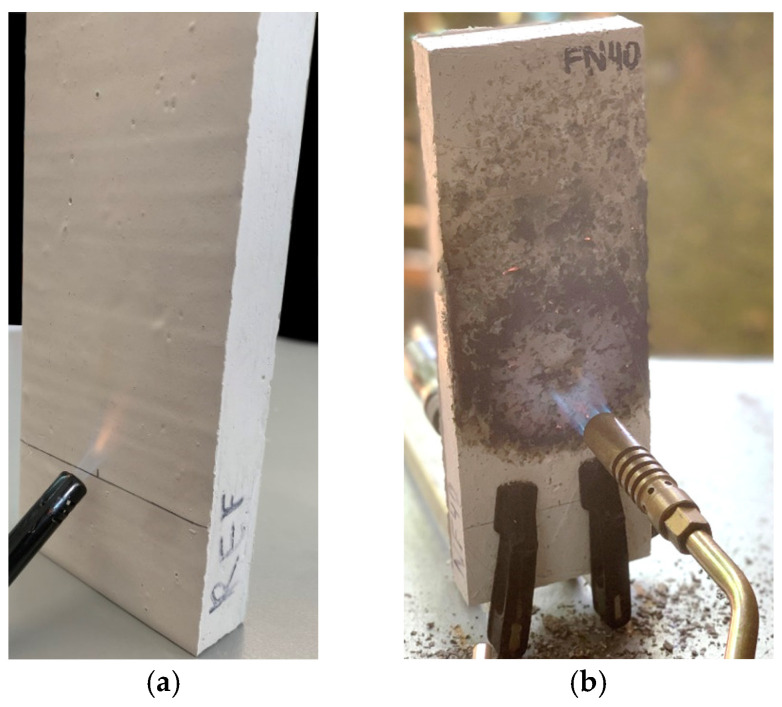
Fire behavior tests: (**a**) small ignition test; (**b**) torch test.

A single-flame source test was conducted according to ISO 11925-2:2020 [33]. A burner tilted at 45° with a flame length of 20 mm was moved horizontally until the flame reached the pre-set contact point on the 25 × 9 × 2.5 cm^3^ samples (Figure 5a). The flame was applied for 15 s to each sample, as recommended by the cited standard. Afterward, we observed whether ignition occurred within 20 s, and if it occurred, the flame height was noted; we also observed if there were any flaming droplets.

A torch test was also performed using the same samples after the single-flame source test. A blow torch, fed by butane gas, was used 5 cm away from the samples for 15 min (Figure 5b), with this being a similar method to that previously used by other researchers [34,35]. Additionally, the temperature after exposure to the fire of the sample surface on the non-exposed face (NEF) was measured and recorded using type K thermocouples and a Pico Technology data logger (Cambridgeshire, England) and Picolog software (version 6.2.13). On the other side of the sample, the temperature of the exposed face (EF) after the test was measured with an HXT digital thermometer (Bihar, India).

#### 2.3.4. Life Cycle Assessment

**Figure 6:**
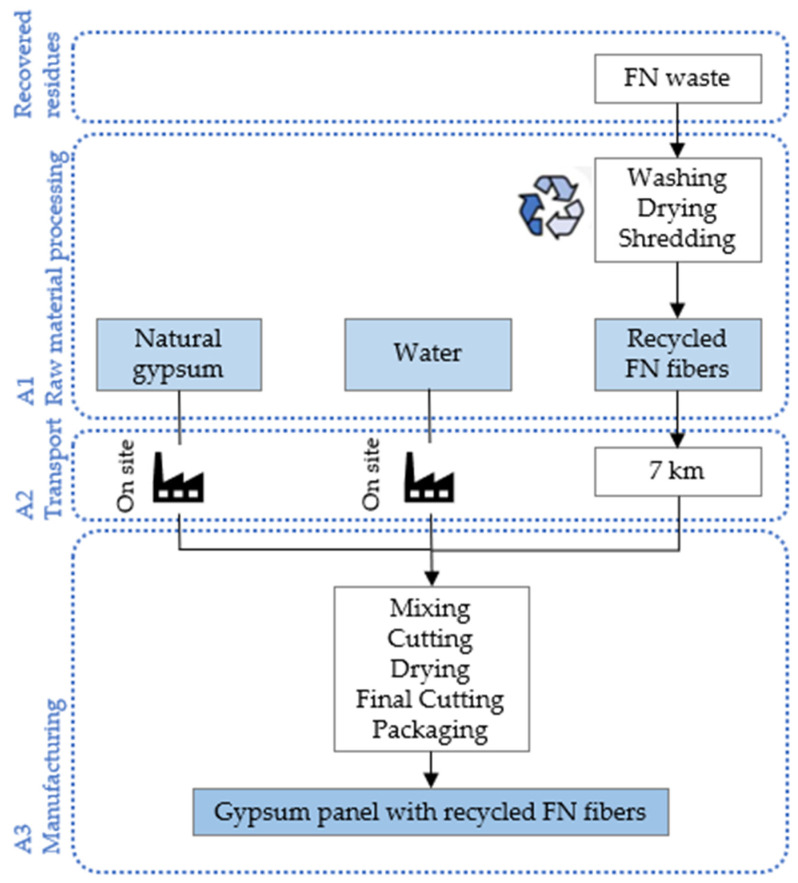
System boundaries of the gypsum panel production process considered for the LCA.

A life cycle assessment (LCA) was carried out according to ISO 14040:2006 [36] and ISO 14044:2006 [37], in which the phases of an LCA are presented: the definition of the objective and scope, the life cycle inventory (LCI) and the impacts assessment (LCIA). The aim of this analysis was to evaluate the impacts associated with the use of recycled FN fibers to manufacture precast gypsum panels for LSF partition walls, considering a functional unit of 1 m^2^ and the production boundaries (cradle-to-gate) shown in Figure 6.

For the LCI, the environmental product declarations for commercial construction gypsum and energy sources were considered, as well as the recycling process for FN waste [15]. It was assumed that the collection and processing of FN waste was conducted at the port of Figueira da Foz (Portugal), 7 km away from the gypsum panel manufacturing facility. Furthermore, gypsum powder and water were supplied by the same facility. Therefore, no impacts related to transport were considered for either material. An inventory of the materials and transportation required to produce each 1 m^2^ composite panel is shown in Table 4, and an inventory of the processes and energy consumption involved is shown in Table 5.

The LCIA was used to quantify the amount of non-renewable energy required for production as the Abiotic Depletion Potential of Fossil Fuels (ADP_ff) and the potential environmental impacts listed in Table 6, using the CML-IA baseline V3.10 method.

## 3. Results and Discussion

### 3.1. Mechanical Properties

**Figure 7:**
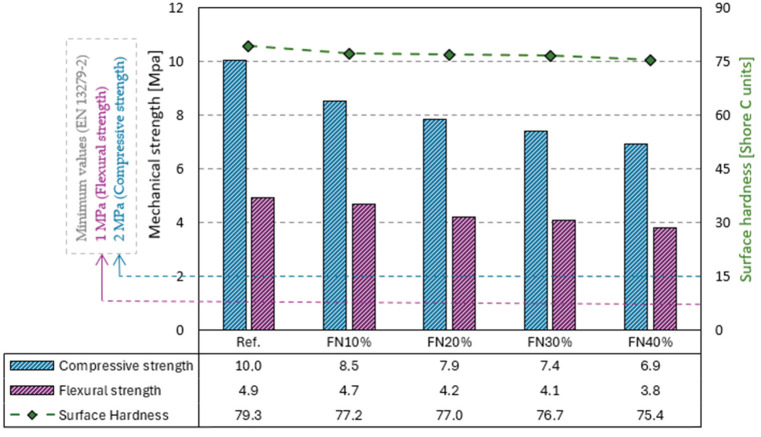
Results of compressive and flexural strength and surface hardness tests.

The results of the compressive and flexural strength and surface hardness tests are presented in Figure 7. The partial replacements of gypsum with recycled FN fibers caused a progressive decrease in these properties.

The surface hardness was the least impacted by the recycled FN fibers’ addition, presenting an almost flat line on the graph. The composite for which we obtained the lowest value was FN40%, with a reduction of 5% compared to the value for the reference one. A similar result was obtained in studies of gypsum matrixes containing other recycled fibers, as an 8.5% decrease was found with PE [38], and a more significant reduction of 22% was found with MW and a graphite polystyrene waste solution (GPWS) [32].

On the other hand, the incorporation of recycled FN fibers had more influence on the compressive and flexural strengths of the developed composites. FN40% also presented the lowest values, 23% and 31%, which were smaller than those of the matrix without the fibers. However, despite the strength reductions, all the composites achieved at least three times the standardized minimum values, 2 MPa for the compressive strength and 1 MPa for the flexural strength [20]. This indicates a good interaction between the matrix and the recycled FN fibers, which is further discussed in light of the microscopic analysis.

A decrease in both properties has been reported by other researchers, as well. Merino et al. achieved reductions of 58% (compressive) and 56% (flexural) in gypsum matrixes with expanded polystyrene (EPS) and extruded polystyrene (XPS) waste fibers incorporated [39]. Nonetheless, better performances have also been reported. Pedreño-Rojas et al. achieved increases of 8% and 13% in the compressive and flexural strengths, respectively, with polycarbonate waste [40], and Romero-Gómez et al. obtained a flexural strength with recycled PP fibers 19% higher than that of the reference, although the compressive strength decreased [41]. Both studies examined gypsum composites.

**Figure 8:**
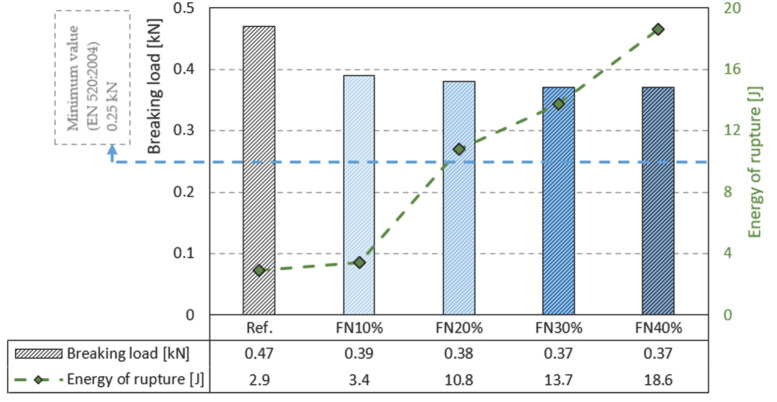
Results of tests of plates’ flexural strength and impact hardness.

Figure 8 presents the results of the tests of the plates’ flexural strength and impact hardness.

Once again, the addition of recycled FN fibers made the flexural strength of the gypsum plate specimens decrease. The composites with 30% and 40% fibers had the lowest value, presenting a decrease of 21% in relation to the matrix without fibers. Nevertheless, the minimum value of 0.25 kN [24] was achieved in all the cases. These results align with those reported in other related studies, e.g., in which expanded polyethylene (EPE) from packaging waste [8] was mixed into gypsum composites.

Unlike the previous results, when evaluating the impact hardness, the energy of rupture showed a great increase. FN10% was a little below the rising trend line, but FN20%, FN30% and FN40% achieved 267%, 367% and 533%, respectively, higher energies of rupture. This demonstrates that the recycled FN fibers further improved the impact hardness of the gypsum panels.

**Figure 9:**
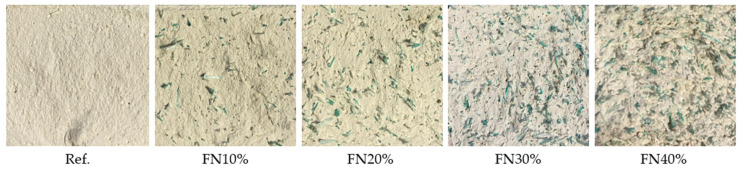
Cross-section images of the samples after the flexural strength test.

**Figure 10:**
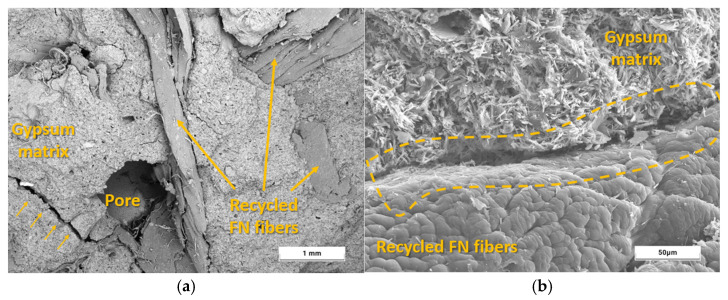
SEM analysis for FN40% sample: (**a**) ×50 magnification; (**b**) ×1000 magnification.

Figure 9 shows cross-section images of the developed composites after the compressive strength test. Macroscopically, a homogeneous distribution of the recycled FN fibers can be noticed, without agglomerations. This became more evident in the SEM images, such as that shown in Figure 10 of the composite with the highest content of recycled material (FN40%), and the surface texture of the original composite was never modified. FN40% was the composite that presented lower strengths in the mechanical tests, so it is important to know its microstructure and how the recycled fibers added to the original gypsum matrix affect it.

Figure 10a provides a general image of the FN40% composite, showing the homogeneous distribution of the fibers in the gypsum matrix during the mixing process. As highlighted by Romero-Gómez et al. in their study, these recycled fibers show good adhesion to the gypsum material because of their surface roughness, caused in part by the wear experienced during the useful life of these FNs and their exposure to UV rays [11]. Also, fiber agglomerations when fibers are added in large quantities can introduce additional porosity into the composites and cause localized defects such as micro-cracks, which increase the susceptibility of the specimen to failure [42]. Figure 10b shows in detail the interfacial transition zone (ITZ) between the fiber and the matrix. The characteristic acicular morphology of the gypsum composites associated with the dihydrate (DH, CaSO_4_·2H_2_O) crystals [21] and the surface roughness of the recycled FN fibers used can be observed. Furthermore, in agreement with other investigations using recycled fibers, the DH crystals precipitated during setting around the reinforcing material [43].

Summarily, the incorporation of recycled FN fibers in the developed composites promoted great mechanical properties with strength values above the current standardized requirements. The impact hardness must be highlighted, as the energy of rupture of the composite with 40% fibers was five times higher than that of the plate made with gypsum and water only. After SEM analysis, it could be verified that the good properties achieved were due to the recycled FN fibers’ great distribution and adhesion to the matrix, proving to be beneficial when included in gypsum panels.

### 3.2. Hygrothermal Properties

**Figure 11:**
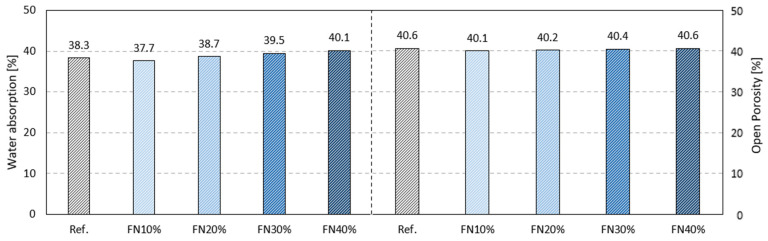
Results of water absorption and open porosity tests.

The results of the water absorption and open porosity tests are shown in Figure 11. It was observed that both properties were minimally influenced by the incorporation of recycled FN fibers in the gypsum matrix, although there was a slight tendency toward increasing water absorption. FN40% absorbed more water than the other composites and 5% more than the reference, while its open porosity remained the same. This behavior has been observed in other studies with gypsum replacements with polymeric waste fibers. Li et al. reported a water absorption increase of 13% with the addition of polyvinyl alcohol fibers [44], and Balti et al. reported that the water absorption almost doubled (96%) when fibers of EPS, polyester and paper waste were incorporated in the same gypsum sample [45].

Regarding the open porosity, FN10% and FN20% only achieved a value 1% less than that of the reference. However, the addition of fibers could increase the hygroscopicity of gypsum composites due to the tendency for the formation of small-diameter pores and connections between them [32]; thus the non-significant variation in this property with the addition of recycled FN fibers can be considered positive.

**Figure 12:**
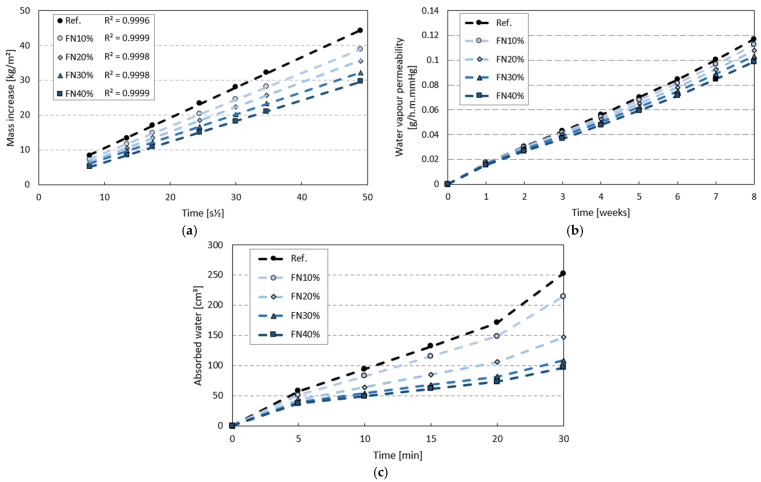
Results of (**a**) water absorption by capillarity, (**b**) water vapor permeability and (**c**) water permeability tests.

The results of water absorption by capillarity, water vapor permeability and water permeability tests are shown in Figure 12. Similar behavior can be observed in all these results, namely a decreasing trend as the incorporation rate of recycled FN fibers increased.

The greatest reduction obtained in the water absorption by capillarity test was in the NF40% composite, with a 33% lower mass increase than that of the gypsum matrix without fibers. Other studies carried out have achieved similar reductions of 34% and 40% with recycled fibers of EPE [8] and EPS [32], respectively. Thus, considering that the increase in the absorption (Figure 11) was very slight (+1.8%) and the open porosity was exactly the same (Ref. and FN40%), it can be concluded that recycled FN fibers, given their polymeric nature (nylon), block the water capillary rise, as expected. Furthermore, due to the structure of these specific nylon multifilament FNs, they can absorb some water within the composite, i.e., between their multiple filaments.

**Figure 13:**
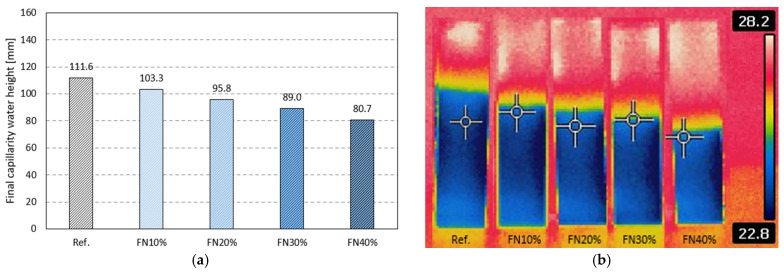
Capillarity water heights: (**a**) final measurements and (**b**) thermographic photo.

Moreover, Figure 13 shows the capillarity water height in each sample after the test, from which we can see lower water levels with higher percentages of recycled FN fibers. The height for FN40% was 28% lower than that of the reference, which corroborates the evaluation of Vidales-Barriguete et al., who considered plastic waste suitable for use in gypsum composite materials when applied in building rooms exposed to water [46]. When fibers are introduced into a plaster composite, the matrix exhibits greater interfacial porosity, which facilitates water penetration [47], as can be observed in Figure 10a showing the SEM analysis. This effect may lead to higher total water absorption in the fiber composites, where the total mass of the absorbed water increases when the specimen is immersed. On the other hand, a larger pore diameter does not necessarily imply greater capillary water absorption, in accordance with Jurin’s Law [48]. Similarly, the incorporation of FN fibers could interrupt the continuity of the capillary pores in the matrix, showing behavior similar to that presented in Figure 12, as reported in other studies [49].

The addition of recycled FN fibers to gypsum composites reduced their water vapor permeability, as seen in Figure 12b. After an 8-week cup method test, the lowest value was obtained for FN40%, 15% lower than that for the matrix without any additions. With gypsum composites incorporating dissolved EPS, Zaragoza-Benzal et al. achieved the same reduction of 15% in the water vapor permeability [21]. Therefore, other researchers have reported similar behavior, including decreases of 37% and 41% with PE waste [38] and plastic cable waste [46], respectively, incorporated in gypsum matrixes.

An improvement in the water resistance of the composites with recycled FN fibers was also observed in the water permeability test (Figure 12c). After fast water penetration in the first 5 min of the test for all the samples, because they were dried, the absorption rate decreased and stabilized up to 20 min, then, due to possible saturation, the rate went up again. This behavior was noticed in all the tested composites, although the volume of absorbed water was lower in those with more recycled FN fibers. Again, we obtained the lowest value for FN40%, achieving a reduction of 62% in comparison to that of the reference. Romero-Goméz et al. also studied the water permeability of gypsum composites incorporating plastic waste and reported decreases of 48% and 42% with recycled PP and PA6 fibers [9], respectively, the latter being the same kind of material as the recycled FN fibers used in this research.

**Figure 14:**
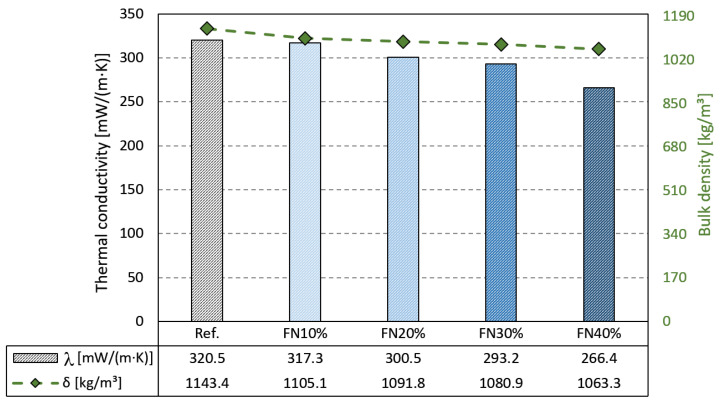
Thermal conductivity and bulk density results.

Figure 14 depicts the results of the thermal conductivity and bulk density tests. As seen in the presented results of the previous tests (mechanical and water properties), the thermal conductivity and bulk density also followed a declining trend as the incorporation rate of recycled FN fibers increased.

Regarding the bulk density, the FN40% composite had a bulk density of 1063 kg/m^3^, representing a decrease of 7% in respect to that of the reference one. This was different to the results presented in another study employing recycled FN fibers, in which a slight increase was noticed in the bulk density of a gypsum composite with a fiber content of 2.5% by mass [50]. This disagreement could be explained by the different w/g ratios and lengths of fibers used. While the recycled FN fibers used in this study had an average length of 6 mm and the w/g ratio of all the samples was 0.7, the average length in the cited study was 20–25 mm and the w/g was 0.55. On the other hand, more significant reductions in the bulk density were achieved in studies with gypsum composites containing recycled EPE [8] and PE [38] fibers.

Compared to the matrix without fibers (320.45 mW/m·K), FN40% presented better thermal performance with 17% lower thermal conductivity (266.35 mW/m·K), due to the low conductivity of plastic materials [9]. As the recycled FN fibers have 75% lower thermal conductivity compared to the reference, they were responsible for reducing the heat conductivity of the composites developed containing fibers. These results support the use of recycled FN fibers as an addition to gypsum panels for use in LSF partition walls. A similar decrease of 21% was obtained in gypsum composites containing a GPWS [32].

**Figure 15:**
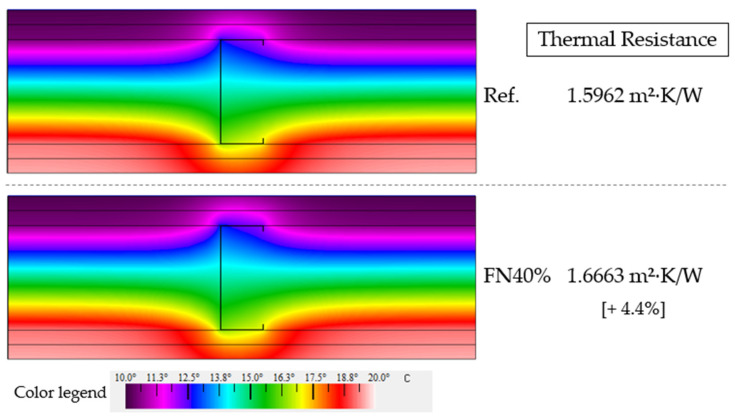
THERM simulation of thermal performance of LSF partition wall.

The THERM simulations performed for an LSF partition wall with the Ref. and FN40% are presented in Figure 15. Although the temperatures in the cross-section areas were similar, the thermal resistance of the set with panels with 40% recycled FN fibers increased by 4.4% compared to that of the set with the reference panels. Thus, the composite developed in this study could improve the thermal performance of buildings, and other cross-section configurations of LSF partition walls must be analyzed to search for even greater results in future studies. With this same configuration, Ferrández et al. achieved an increase of 20.3% in the thermal resistance with gypsum panels containing EPE [8].

### 3.3. Fire Behavior

**Figure 16:**
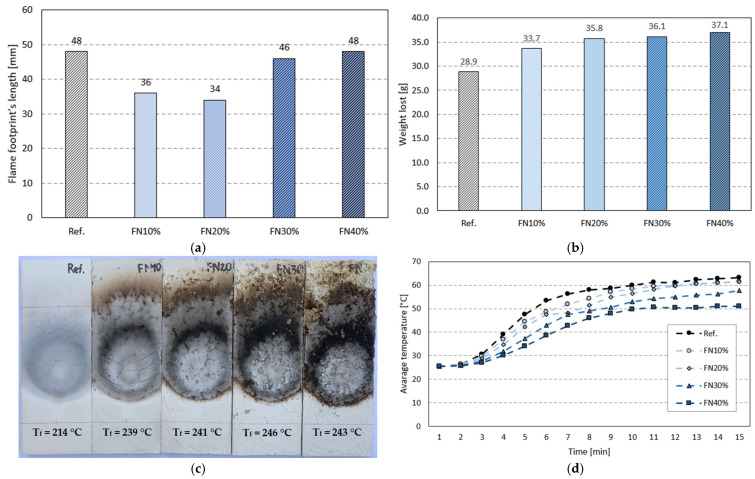
Results of fire tests: (**a**) small ignition footprints; (**b**) weight lost from samples after torch test; (**c**) EFs and temperatures after torch test; (**d**) average temperature of NEFs during torch test.

The results of the fire tests are shown in Figure 16. None of the samples ignited, nor did any flaming particles occur, maybe because gypsum is not a flammable material and the single-flame test’s duration was only 15 s, not enough time for ignition. The flame tip did not reach more than 150 mm above the application point in any case. In fact, the flame footprint’s length did not even get close to that value, as is visible in Figure 16a. These footprints were minimal and nearly imperceptible, just like those reported by Álvarez et al. [51].

Regarding the torch test, the specimens lost more mass the more recycled FN fibers they had, as seen in Figure 16b. The mass loss of FN40% was 28% more than the reference’s, and its EF was the most affected by the fire, presenting more disintegration, as shown in Figure 16c. All the samples showed crack patterns and circular combustion stains; once again, these were more relevant the higher the recycled FN fiber content was, due to the ignition of the fibers and microstructural and mineral alterations in the gypsum matrixes [34].

It can be observed in Figure 16d that the temperature of the samples’ NEFs changed in similar phases during the torch test. In the first half, an increasing rate was noticed, followed by a still increasing but more stable rate until the end. Figure 16d also shows that, in spite of the effects of fire and the temperature being bigger at the EFs, the final temperature at the NEFs decreased with the addition of recycled FN fibers. The heat propagation through the composites was lower in those with a greater fiber content, opposite to our expectations based on past studies [35].

Despite the greatest mass loss being achieved in FN40%, the temperature of FN40%’s NEF after the test decreased by 19% compared to that of the matrix without any additions. This confirms the results of the thermal conductivity test and THERM simulation. The lower thermal conductivity of the FN40% sample reduced the heat conduction through the sample and lowered the temperature of the NEF, even with a greater mass loss. This loss was due not primarily to the loss of gypsum mass, but mostly because the fishing net fibers are combustible. Therefore, having a higher fiber content, more material was burned, and consequently, as expected, a higher mass loss was obtained.

### 3.4. Life Cycle Assessment

**Figure 17:**
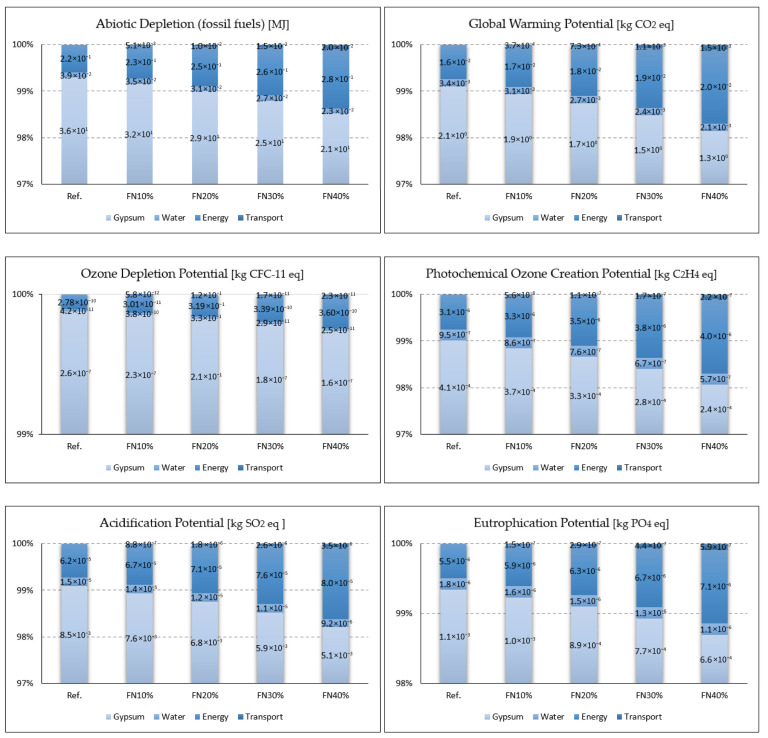
Contribution of each production process to the potential environmental impacts of the developed gypsum composite panels (1 m^2^).

The results of the LCA are presented in Table 7, and the contribution of each process in producing 1 m^2^ of gypsum panels with recycled FN fibers to the potential environmental impacts are shown in Figure 17.

The gypsum powder production process was responsible for more than 98% of the total values obtained for all the impact categories analyzed. Furthermore, its contribution was more than 99% for the ODP, making the other contributions negligible. Romero-Gómez et al. also obtained the highest impacts from the raw material supply stage, which corresponds to the process of manufacturing commercial gypsum, compared to the minimal impact from the FN waste recycling process [9]. This explains why the impact reductions correlated with the percentages of gypsum’s replacement with the fibers in all the categories. The FN10% sample’s calculated impact was 10% less than the reference’s, and the FN20% sample’s was 20% less and the FN30 sample’s and FN40% sample’s were 30% and 40%, respectively, less.

Despite the positive results regarding the environmental impacts for the incorporation of recycled FN fibers in gypsum composites used for the construction of panels, carrying out an LCA of recycled gypsum containing those fibers would be important to verify its greater environmental performance in regard to the circular economy and use of more sustainable materials.

## 4. Conclusions

In this study, the influence of incorporating recycled FN fibers into gypsum composites on their mechanical, hygrothermal, fire and environmental performances was evaluated. The main highlights are listed below:

- The composites developed containing recycled FN fibers presented both compressive and flexural strengths smaller than those of the reference without fibers, following a decreasing trend line as the fiber–gypsum ratio increased. However, all the samples achieved the current standardized minimum strengths. The same behavior was obtained in the flexural strength of plates.
- Regarding the impact hardness, FN40% had an energy of rupture 533% higher than that of the reference. Additionally, the SEM analysis allowed us to notice the fibers’ great distribution and adhesion to the gypsum matrix, which legitimated the great mechanical properties achieved.
- The water absorption and open porosity were not significantly influenced by the incorporation of recycled FN fibers in the composites. On the other hand, a decreasing tendency was also observed in the other hygrothermal properties. The water absorption by capillarity, water vapor permeability and water penetration of the FN40% composite were 33%, 15% and 62%, respectively, less than those of the reference. These results supported the suitability of gypsum panels containing recycled FN fibers for use in wet rooms in buildings.
- The recycled FN fibers decreased the bulk density and thermal conductivity of the gypsum composites by 7% and 17%, respectively, when it came to the FN40% sample. These reductions support the addition of recycled FN fibers to gypsum panels for use in LSF walls as partitions or even facades.
- In fact, the use of FN40% composites in a LSF partition wall increased its thermal resistance by 4.4% in reference to traditional gypsum plasterboards (Ref.) with the same thickness.
- Although the mass loss of the FN40% sample was the biggest after the torch test, the temperature of its NEF was the smallest. This means that the recycled FN fibers acted against heat propagation, which validates the lower thermal conductivity measured in the laboratory test and the higher thermal resistance predicted by the THERM simulation.
- The manufacturing process for the gypsum powder was the most responsible for the environmental impacts across all the categories analyzed in the LCA. Thus, the impact of the recycling process for the FN waste became negligible and the reductions in the impact categories were proportional to the gypsum replacements with the recycled FN fibers. Thereby, FN40% had 40% less environmental impacts than the reference in all the categories.

Despite the excellent results obtained with the gypsum composites with recycled FN fibers in comparison to those for the matrix without fibers, improvements still can be made to achieve better properties. Indeed, future studies must evaluate the incorporation of recycled FN fibers in recycled gypsum mixtures to further improve their thermal and environmental performances. Additionally, more advanced microstructural characterization, such as XRD analysis, is recommended.

## Figures and Tables

**Table 1:** Evaluated samples’ compositions.

Sample	Gypsum [g]	Water [g]	FN Fibers [g]
Ref.	1000	700	0.0
FN10%	900	630	16.7
FN20%	800	560	33.3
FN30%	700	490	50.0
FN40%	600	420	66.7

**Table 2:** Description of the variables in Equations (3) and (4).

Variable	Description	Unit
*P*	Water vapor permeability	g/(m.s.mmHg)
*W*	Water vapor permeance	g/(m^2^.s.mmHg)
Δ*m*	Mass variation	g
Δ*t*	Time between weighings	s
Δ*p*	Water vapor pressure difference across sample	mmHg
*A*	Sample area	m^2^
*d*	Sample thickness	m

**Table 3:** Thickness (t) and thermal conductivity (λ) of the materials used in the reference LSF wall.

Material	*t* [mm]	*λ* [W/(m·K)]	Ref.
Gypsum panels	12.5	Section 3.2	Measured
Mineral wool	90.0	0.035	[32]
Steel studs (C90 × 37 × 5 × 0.6)	90.0	50	[32]

**Table 4:** Inventory results for material and transport inputs for each 1 m^2^ composite panel.

Material Inputs	Ref.	FN10%	FN20%	FN30%	FN40%	Transportation [km]
Gypsum powder [g]	16.3	14.6	13.0	11.4	9.8	-
Water [g]	11.4	10.3	9.1	8.0	6.8	-
FN fibers [g]	-	0.3	0.5	0.8	1.1	7.0

**Table 5:** Inventory of processes and energy consumption involved in the production of each composite panel.

Stage	Process	Power	Consumption per ton	Units	Ref.
FN recycling	Washing, drying and shredding	Electricity	20	kWh	[9]
Panel production	Mixing	Electricity	0.8	kWh	[9]
	Molding	Electricity	2.78	kWh	[9]
	Drying	Electricity	1.11	kWh	[9]
	Packaging	Electricity	0.02	kWh	[9]

**Table 6:** Indicators considered in the LCIA conducted and their units.

Indicators	Units
Abiotic Depletion Potential of Fossil Fuels (ADP_ff)	MJ
Global Warming Potential (GWP)	kg CO_2_ eq
Ozone Depletion Potential (ODP)	kg CFC-11 eq
Acidification Potential (AP)	kg SO_2_ eq
Eutrophication Potential (EP)	kg PO_4_ eq
Photochemical Ozone Creation Potential (POCP)	kg C_2_H_4_ eq

**Table 7:** Potential environmental impacts of 1 m^2^ of gypsum panels containing recycled FN fibers.

Environmental Impact Category	Ref.	FN10%	FN20%	FN30%	FN40%
ADP_ff [MJ]	3.6 × 10^1^	3.3 × 10^1^	2.9 × 10^1^	2.5 × 10^1^	2.2 × 10^1^
GWP [kg CO_2_ eq]	2.1 × 10^0^	1.9 × 10^0^	1.7 × 10^0^	1.5 × 10^0^	1.3 × 10^0^
ODP [kg CFC-11 eq]	2.6 × 10^−7^	2.3 × 10^−7^	2.1 × 10^−7^	1.8 × 10^−7^	1.6 × 10^−7^
POCP [kg C_2_H_4_ eq]	4.1 × 10^−4^	3.7 × 10^−4^	3.3 × 10^−4^	2.9 × 10^−4^	2.5 × 10^−4^
AP [kg SO_2_ eq]	8.5 × 10^−3^	7.7 × 10^−3^	6.9 × 10^−3^	6.0 × 10^−3^	5.2 × 10^−3^
EP [kg PO_4_ eq]	1.1 × 10^−3^	1.0 × 10^−3^	8.9 × 10^−4^	7.8 × 10^−4^	6.7 × 10^−4^

## Data Availability

The original contributions presented in this study are included in the article. Further inquiries can be directed to the corresponding author.

## Abbreviations

The following abbreviations are used in this manuscript:

FNs	Fishing nets
LSF	Lightweight steel-framed
PP	Polypropylene
PE	Polyethylene
PA	Polyamide
LCA	Life cycle assessment
SEM	Scanning electron microscopy
MW	Mineral wool
NEF	Non-exposed face
EF	Exposed face
LCI	Life cycle inventory
LCIA	Life cycle impact assessment
ADP_ff	Abiotic Depletion Potential of Fossil Fuels
GWP	Global Warming Potential
OPD	Ozone Depletion Potential
AP	Acidification Potential
EP	Eutrophication Potential
POCP	Photochemical Ozone Creation Potential
GPWS	Graphite polystyrene waste solution
ELT	End-of-life tire
EPS	Expanded polystyrene
XPS	Extruded polystyrene
